# Explainable artificial intelligence identifies circulating microRNA candidate biomarkers for liquid biopsy-based pancreatic cancer detection

**DOI:** 10.3389/frai.2026.1810551

**Published:** 2026-10-21

**Authors:** Saurav Mandal, Md Aamir Ansari, Waribam Pratibha Chanu

**Affiliations:** 1 Metabolomics, Proteomics and Imaging Division, Indian Council of Medical Research (ICMR), Dibrugarh, India; 2 Department of Bioinformatics, Central University of South Bihar, Gaya, India; 3 School of Vocational Studies and Applied Sciences, Gautam Buddha University, Greater Noida, Uttar Pradesh, India

**Keywords:** biomarker discovery, c-microRNAs, differential expression, explainable AI, machine learning, pancreatic cancer, SHAP

## Abstract

**Introduction:**

Pancreatic cancer (PC) remains a highly lethal malignancy due to asymptomatic progression, late presentation, and lack of reliable minimally invasive liquid biopsy biomarkers for early detection. Circulating microRNAs (c-miRNAs) are promising clinical indicators owing to their stability in body fluids and regulatory influence on oncogenic pathways.

**Methods:**

This study presents an integrated computational framework combining large-scale serum miRNA expression profiling with differential expression analysis, machine learning (ML), and explainable artificial intelligence (XAI). Five publicly available GEO datasets comprising 1,101 serum samples were analyzed through preprocessing, including normalization, missing-value imputation, and ComBat-based batch correction. A Bayesian stacked ensemble classifier integrating XGBoost, Random Forest, SVC, Naïve Bayes, and k-NN was optimized using SMOTE for class imbalance and constrained weight minimization via SLSQP.

**Results:**

Gene expression analysis identified 148 significantly dysregulated miRNAs (|log2FC| ≥ 2, adj. *p* < 0.05). The model achieved excellent predictive performance (AUC = 0.97; accuracy = 97.28%; sensitivity = 94%; specificity = 99%). SHAP-based XAI interpretation highlighted 20 miRNAs as the most influential predictors, including hsa-miR-125a-3p, hsa-miR-1290, hsa-miR-614, and hsa-miR-497-5p. Pathway enrichment using DIANA-miRPath and miRDB confirmed their involvement in key oncogenic pathways (MAPK, CDK, TP53).

**Discussion:**

As a retrospective computational study, these findings represent computationally prioritized candidate biomarkers requiring validation in independent external cohorts and experimental approaches before clinical application. The proposed pipeline demonstrates a transparent, reproducible approach for biomarker discovery and validates the clinical promise of circulating miRNAs in minimally invasive liquid biopsy-based early detection of pancreatic cancer.

## 1 Introduction

Pancreatic cancer (PC) is widely recognized as one of the deadliest malignancies and continues to pose a significant clinical challenge because of its aggressive progression, diagnostic complexity, and poor survival outcomes (Khan and Saraya, 2023; Rachagani et al., 2015). PC is responsible for about 4.7% of all cancer-related deaths, despite accounting for only 2.6% of all cancers globally. Nearly 500,000 new cases of cancer are reported worldwide each year, according to recent statistics, indicating a steady increase in the disease’s incidence (Ilic and Ilic, 2022). Due to the high probability of diagnosis at advanced stages and the asymptomatic presentation typical of early-stage disease, the overall five-year survival rate is still significantly limited at about 6%. Most cases of PC arise from the exocrine duct epithelium of the pancreas and often progress via precursor lesions before evolving into invasive carcinoma (Chi et al., 2023).

Nearly 70% of pancreatic tumors start in the head region, and they often show no obvious early symptoms. Abdominal pain, dark urine, anorexia, exhaustion, and obstructive jaundice are typical symptoms, though they are nonspecific and can also be present in other benign or inflammatory conditions. Moreover, severe cachexia greatly increases mortality and is often linked to pancreatic cancer. The fact that inflammatory diseases like acute or chronic pancreatitis can closely resemble PC on clinical and radiological evaluation further complicates early diagnosis. Confirmation of malignancy often requires invasive procedures, which include endoscopic ultrasound-guided fine needle aspiration (EUS-FNA), percutaneous biopsy, and surgical pathology. Cytology-based diagnostic accuracy is limited by sampling-related challenges, inflammation, or tissue necrosis, and invasive sampling carries risks such as pancreatic fistula formation (Liu et al., 2023; Terada et al., 2019). Given these diagnostic limitations, the search for sensitive, minimally invasive liquid biopsy biomarkers has intensified.

Circulating microRNAs (c-miRNAs) have gained considerable attention as potential biomarkers because of their biological significance, stability, and detectability in serum and other biological fluids. MicroRNAs are small, non-coding RNA molecules that regulate gene expression after transcription in many different organisms (Panagioti et al., 2018). They are typically between 18 and 25 nucleotides long. The research teams of Ambros and Ruvkun first identified them in Caenorhabditis elegans in 1993, which paved the way for a new area of study in molecular regulation and gene expression control. Pri-miRNA transcription initiates the miRNA maturation process, which proceeds through pre-miRNA intermediates and concludes with mature miRNA. Mature miRNAs typically bind to the 3′ untranslated regions of target mRNAs, leading to translational repression or mRNA degradation, although they can also participate in transcriptional activation under specific contexts (O’Brien et al., 2018).

The rapid expansion of miRNA research led to the development of miRBase, an online repository that catalogs miRNA sequences and annotations (Griffiths-Jones, 2006). Its latest version contains more than 38,000 hairpin precursors and over 48,000 mature sequences from 271 species, including 2,654 miRNAs in the human genome (Kozomara et al., 2019). miRNAs play critical roles in cell growth, metabolism, cell signaling, and differentiation, and miRNAs are predicted to modulate the expression of over 30% of human mRNA (Zen and Zhang, 2012). Recent advances in transcriptomic analysis, including reference-free approaches, further highlight the importance of integrative computational frameworks in cancer biomarker discovery (Eralp and Sefer, 2024).

c-miRNAs have gained particular interest because they are detectable in serum, plasma, saliva, cerebrospinal fluid, breast milk, urine, tears, peritoneal fluid, and other accessible fluids (Brase et al., 2010; da Silveira et al., 2012; Gallo et al., 2012; Zhou et al., 2011). These molecules are remarkably stable and can withstand harsh physical and chemical conditions, which include repeated freeze-thaw cycles, long-term storage, and fluctuations in pH and temperature. Their stability and differential expression patterns in health and disease have positioned them as attractive minimally invasive liquid biopsy biomarkers for detecting cancer, prognosis, and therapeutic monitoring. Moreover, c-miRNAs participate in intercellular communication and can influence tumor microenvironment dynamics, which further highlights their biological significance (Hergenreider et al., 2012; Wang and Chen, 2014).

Despite the substantial evidence supporting c-miRNAs diagnostic value, inter-study variability, dataset heterogeneity, technical noise, and batch effects make it challenging to translate this potential into clinical application. Complex computational techniques are needed to enable precise feature selection, pattern extraction, and biological interpretation of high-dimensional multi-platform miRNA datasets. Machine learning (ML) algorithms have demonstrated substantial potential for cancer state classification by effectively extracting patterns from high-dimensional omics data. Nevertheless, traditional machine learning methods frequently serve as “black box” models that lack mechanistic understanding. By identifying significant molecular contributors, explainable artificial intelligence (XAI), such as Shapley Additive Explanations (SHAP), provides an effective approach that improves interpretability and supports biological validation (Nohara et al., 2019).

In response to these challenges, the present study focuses on the developing an integrated computational pipeline for identifying circulating miRNA biomarkers associated with pancreatic cancer. This approach incorporates preprocessing of five large serum miRNA datasets (Table 1), differential expression analysis, ML based classification, Bayesian stacking for improved prediction accuracy, and SHAP-based interpretability. Additionally, pathway enrichment analysis using DIANA miRPath and miRDB was executed to illuminate the biological significance of the top predictive miRNAs (Griffiths-Jones, 2006; Tastsoglou et al., 2023). By combining statistical rigor with advanced ML and XAI, this framework offers a robust and transparent methodology for minimally invasive liquid biopsy biomarker discovery and contributes toward the development of early detection strategies in pancreatic cancer.

**Table 1:** Detailed information of the GEO datasets of c-miRNAs expression datasets.

GEO accession	Contributor	Platform	Sample (N: PC)	Year	Source
c-miRNAs GSE59856	Kojima et al. (2015)	GPL18941, 3D-Gene Human miRNA V20_1.0.0	(150:100)	2015	Serum
c-miRNAs GSE106817	Yokoi et al. (2018)	GPL21263, 3D-Gene Human miRNA V21_1.0.0	(200:115)	2018	Serum
c-miRNAs GSE112264	Urabe et al. (2019)	GPL21263, 3D-Gene Human miRNA V21_1.0.0	(41:50)	2019	Serum
c-miRNAs GSE113486	Usuba et al. (2019)	GPL21263, 3D-Gene Human miRNA V21_1.0.0	(100:40)	2018	Serum
c-miRNAs GSE124158	Asano et al. (2019)	GPL21263, 3D-Gene Human miRNA V21_1.0.0	(275:30)	2019	Serum

(N, Normal control sample; PC, Pancreatic cancer sample).

## 2 Methods

Multiple cohort serum miRNA data collection, thorough preprocessing, statistical differential expression analysis, supervised machine learning, Bayesian ensemble modeling, explainable artificial intelligence, and biological pathway validation were all included in the current study’s integrative computational framework.

### 2.1 Data acquisition

To identify differences in c-miRNA expression between pancreatic cancer patients and healthy controls, a systematic search was executed within the Gene Expression Omnibus (GEO) for relevant public serum-based datasets. The keywords “pancreatic cancer serum” and “miRNA” were used to filter relevant datasets (Han et al., 2016). Five studies that satisfied the inclusion criteria of serum-based sampling, human subjects, and microRNA profiling were selected. These datasets, namely GSE59856 (Kojima et al., 2015) GSE106817 (Yokoi et al., 2018), GSE112264 (Usuba et al., 2019), GSE113486 (Usuba et al., 2019), and GSE124158 (Asano et al., 2019), were retrieved using the GEOquery package in R, which enabled automated extraction of metadata and expression matrices. Together, the datasets comprised 1,101 serum samples, which represented one of the largest combined c-miRNA cohorts available for pancreatic cancer research. The substantial sample size was essential for both statistical power and ML model generalizability.

Each dataset was generated using high-density microarray platforms such as the 3D Gene Human miRNA V20 and V21 arrays. These platforms capture a broad spectrum of mature human miRNAs, enabling cross-study harmonization. The datasets were recorded between 2015 and 2019, and although profiling technologies were similar across platforms, differences in experimental conditions and preprocessing pipelines were expected. Detailed characteristics, including accession numbers, platforms, sample sizes, and sources, are provided in Table 1. The detailed workflow of the methodology is presented in Figure 1.

**Figure 1:**
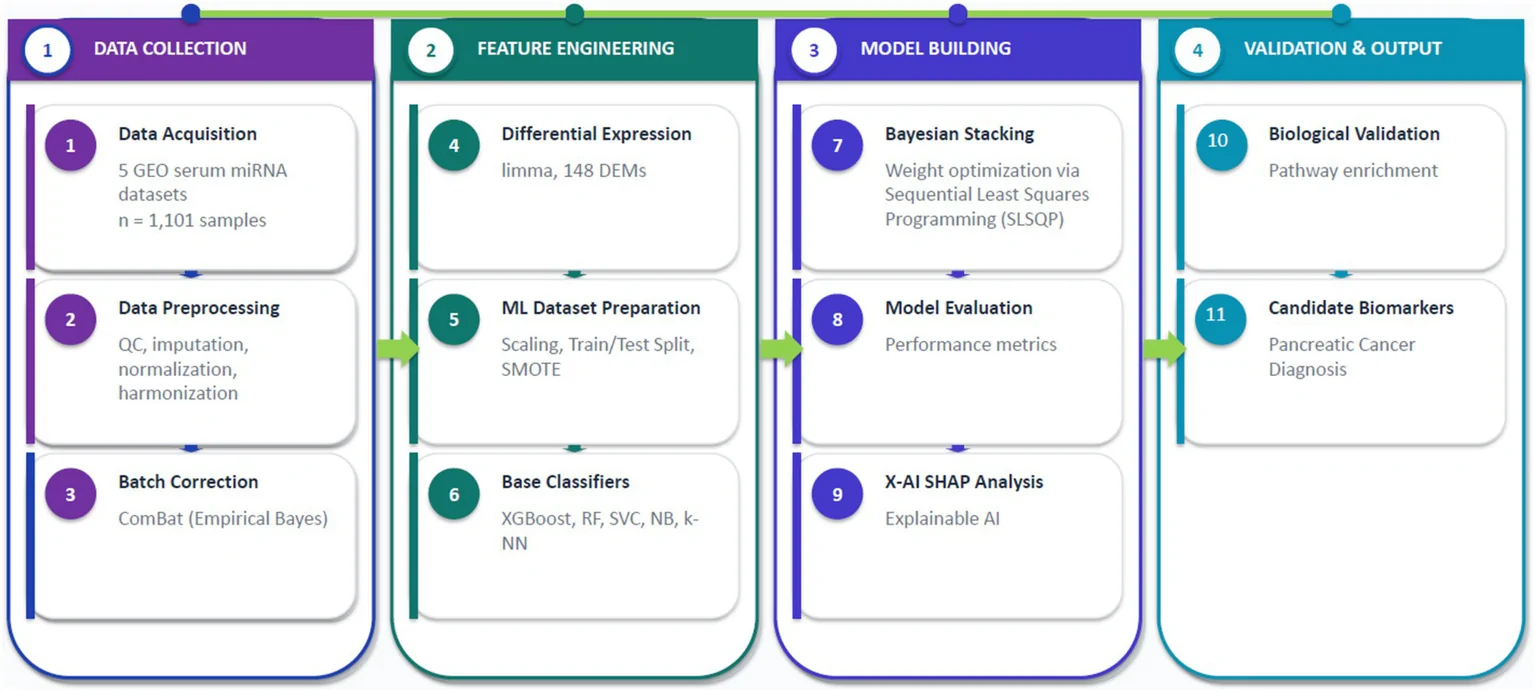
Schematic overview of the proposed computational explainable AI framework for pancreatic cancer biomarker discovery using circulating microRNAs (c-miRNAs).

### 2.2 Data preprocessing and integration

After the acquisition, we imported raw expression files for all datasets into Python for preprocessing. Microarray-generated c-miRNA data can show variability due to technical issues, differences in hybridization efficiencies, and design discrepancies across batches. To ensure the biological consistency of the merged datasets, we implemented a thorough preprocessing pipeline. The initial quality assessment checked for missing values, outliers, and inconsistencies in miRNA naming conventions. We filled in missing values using mean substitution, which is common for high-dimensional data when the missing data is low and random. This method preserved the underlying distribution of expression values and minimized potential distortions during later statistical analysis. After imputing the data, we aligned all datasets by matching miRNA identifiers across studies. Each GEO dataset had its own order and specific platform annotations. Carefully merging the miRNA features was necessary to avoid duplication or mismatches. Once we aligned the datasets, we combined them into two matrices: one for healthy controls and another for pancreatic cancer samples. The GEO Series Matrix files used in this study contain processed expression values generated after platform-specific background correction and signal extraction performed by the original studies. Therefore, our analysis began from normalized expression matrices rather than raw fluorescence intensities. Negative expression values occasionally observed in processed microarray data are expected following background correction and normalization and do not represent biologically negative miRNA expression.

Because the five datasets originated from different laboratories, platforms, and years, significant batch effects were anticipated. Uncorrected batch effects can overshadow true biological signals and lead to spurious results in differential expression and ML models, a problem that has been well documented in genomic studies (Leek et al., 2010). To mitigate this, batch correction using the ComBat algorithm was performed through the pyComBat library in Python. ComBat applies an empirical Bayes framework to stabilize variances and adjust expression distributions across batches while preserving biological variability (Johnson et al., 2007). This correction step ensured that differences in miRNA expression reflected biological distinctions between disease and control groups rather than technical artifacts. Batch corrected matrices for both cancer and control groups were exported for differential expression analysis. All.

### 2.3 Differential expression analysis using Limma

The limma package in R was employed to analyze batch-corrected datasets for identifying miRNAs that exhibited significant dysregulation between pancreatic cancer and control samples (Ritchie et al., 2015). Limma stabilizes variance estimates in high-dimensional microarray data by combining linear modeling with empirical Bayes shrinkage, making it a highly reliable approach for miRNA differential expression analysis. In this study, differential expression analysis using limma was performed on the full dataset prior to ML modeling as a biological feature discovery step, rather than as a predictive feature selection procedure. The primary objective of this step was to identify statistically significant and biologically relevant miRNAs associated with pancreatic cancer. This approach is consistent with standard practices in omics-based biomarker studies, where statistical filtering is applied prior to downstream predictive modeling to reduce dimensionality and enhance interpretability.

Prior to model fitting, both PC and healthy datasets underwent log2 transformation to normalize the distributions of microarray intensities. Gene-wise linear models were then fitted by limma to estimate log changes across all c-miRNAs. To address hypothesis testing adjusted *p*-values were calculated using the Benjamini-Hochberg false discovery rate procedure (Patterson et al., 2010). miRNAs were deemed dysregulated if they exhibited an absolute log2 fold change of no less than two along, with an adjusted *p*-value below 0.05 (Zhu et al., 2025). These thresholds ensured biological relevance and minimized false positives. The analysis identified 148 significant differentially expressed miRNAs (DEMs). Visual exploration was performed through volcano plotting and principal component analysis, which demonstrated strong differentiation between healthy and cancer samples, suggesting strong discriminatory potential of c-miRNA expression profiles.

### 2.4 ML classification pipeline

#### 2.4.1 Data preparation

The set of 148 DEMs was used to construct the ML input dataset. The miRNA matrices were transposed to form a sample by feature structure, wherein each row denotes an individual serum sample and each column representing a particular miRNA. Python was selected for its efficient handling of large-scale, high-dimensional datasets and its seamless integration of data preprocessing, machine learning, and explainable AI workflows. Detailed information regarding all Python packages and software versions used in this study is provided in Supplementary Figure S4 to ensure reproducibility. Class labels were assigned as 1 for pancreatic cancer and 0 for healthy controls. Because ML algorithms are sensitive to feature scales, all features were standardized using the StandardScaler function in Python, which transformed each miRNA to have a mean of zero and a standard deviation of one. The dataset was randomly partitioned allocating 80% of data to the training set and 20% to the testing set, ensuring that both subsets retained proportional representation of PC and control samples. Additionally, 3% of the class labels in the training data were intentionally flipped to simulate real world noise and to evaluate the robustness of classifiers in the presence of label uncertainty. To evaluate the robustness of the proposed models under realistic conditions, a small proportion of label noise was introduced into the training data. A 3% label perturbation was introduced as a predefined computational robustness scenario to evaluate the sensitivity of the machine-learning models to a small proportion of potentially incorrect class assignments. The 3% perturbation was not intended to represent an empirically estimated diagnostic misclassification rate for pancreatic cancer. The original GEO-derived disease annotations were retained as the primary biological labels, and the label-noise experiment was considered an auxiliary robustness assessment. The chosen noise level represents a controlled perturbation that is sufficient to assess model stability while preserving the underlying biological signal, which is critical for reliable biomarker discovery. This approach is consistent with established practices in ML robustness studies, where low-level label noise is introduced to evaluate model performance under realistic conditions (Frenay and Verleysen, 2014; Rolnick et al., 2018). Importantly, label perturbation was applied only to the training set, ensuring that the test set remained unbiased and reflective of true model generalization. Also, it is important to note that the input features used for ML were derived from prior statistical analysis and were not selected through model-driven optimization. Therefore, the feature selection process was not influenced by model performance metrics, but rather by biological and statistical significance. ComBat was applied to correct for dataset-specific batch effects, with disease status retained for downstream biological interpretation. No additional clinical covariates were included in the batch-correction model because these variables were not consistently available across the five datasets.

#### 2.4.2 Addressing class imbalance

Although the combined dataset was large, class imbalance existed due to unequal representation of disease and control samples across studies. To correct this imbalance and enhance model performance, The Synthetic Minority Over-Sampling Technique (SMOTE) was utilized on the training dataset (Majyambere et al., 2026). SMOTE generates synthetic samples in the feature space of the minority class, thereby improving classifier sensitivity without duplicating observations (Fernandez et al., 2018). The aggregated dataset exhibited moderate class imbalance due to unequal representation of pancreatic cancer and control samples across cohorts, with an approximate ratio of ~1.4:1. Although not extreme, such imbalance can bias ML models toward the majority class and reduce sensitivity for minority class detection. To mitigate this, the SMOTE was applied exclusively to the training dataset. SMOTE generates synthetic samples in the feature space of the minority class, improving class balance without duplicating observations. The test set was not subjected to oversampling, ensuring unbiased evaluation of model performance.

#### 2.4.3 Model training and ensemble construction

Five diverse ML algorithms were selected as base classifiers. These included Extreme Gradient Boosting (XGBoost) (Chen and Guestrin, 2016), Support Vector Classifier (Hearst et al., 1998; Moguerza and Muñoz, 2006), Random Forest (Breiman, 2001), Naïve Bayes (Zhang, 2004), and k Nearest Neighbors(Guo et al., 2003). Each algorithm offers distinct advantages, such as tree-based robustness (XGBoost, Random Forest), probabilistic modeling (Naïve Bayes), margin-based separation (SVC), and distance-based pattern recognition (kNN). The use of heterogeneous learners increased the likelihood of capturing complementary decision boundaries that may be present in high-dimensional miRNA expression data. The primary modeling objective was to develop a Bayesian stacking framework integrating heterogeneous base classifiers (XGBoost, Random Forest, SVC, Naïve Bayes, and kNN), with model-specific weights estimated by constrained optimization of the combined probabilistic predictions.

Following individual model training, a Bayesian stacking ensemble was constructed to integrate predictions from all five classifiers. Ensemble stacking was performed by optimizing non-negative weights that minimized the negative log likelihood on a dedicated validation subset (Yao et al., 2018). Unlike simple voting or averaging, Bayesian optimization allowed the model to learn optimal contribution weights for each classifier based on their predictive reliability (Brochu et al., 2010). This approach enabled the ensemble to outperform individual learners and reduced the variance associated with overfitting.

To provide a detailed implementation of the Bayesian stacking framework, the dataset was first partitioned into training (80%) and testing (20%) subsets using stratified sampling. The training set was further divided into a stack training subset (80%) and a validation subset (20%) to enable weight optimization. Class imbalance in the training data was addressed using SMOTE prior to model training.

Five base classifiers XGBoost, Random Forest, SVC, Naïve Bayes, and k-NN were trained on the stack training subset. Please refer to Supplementary Table S2 for the parameter settings of all base classifiers used in the Bayesian stacking framework. Each model generated probability estimates for the positive class, which were then evaluated on the validation subset.

The predicted probabilities generated by the individual base classifiers were combined using a weighted linear ensemble. Let $p_{i}(x)$ denote the predicted probability of the positive class produced by the $i^{\mathrm{th}}$ base classifier for an input sample x . The final ensemble probability was computed as:


$$\hat{p}(x)=\sum_{i=1}^{M}w_{i}p_{i}(x)$$

Where M is the total number of base classifiers ( M=5 ), $w_{i}$ denotes the weight assigned to the $i^{\mathrm{th}}$ classifier, and $\hat{p}(x)$ is the final ensemble probability.

The optimal weights were estimated by minimizing the negative log-likelihood (binary cross-entropy) over the validation set:


$$L(w)=-\sum_{j=1}^{N}\log[y_{j}{\hat{p}}_{j}+(1-y_{j})(1-{\hat{p}}_{j})]$$

Where N denotes the number of validation samples, $y_{j}\in \{0,1\}$ is the true class label of the $j^{\mathrm{th}}$ sample, and ${\hat{p}}_{j}$ represents the weighted ensemble probability for that sample. To ensure numerical stability during optimization, the predicted probabilities were clipped to the interval [ε,1−ε] , where $\varepsilon =10^{-15}$ .

The optimization problem was solved using the Sequential Least Squares Programming (SLSQP) algorithm under the following constraints:


$$\sum_{i=1}^{M}w_{i}=1$$

$$0\leq w_{i}\leq 1,i=1,2,\ldots ,M$$

These constraints ensure that the learned weights form a valid convex combination of the individual classifiers, resulting in a probabilistically interpretable ensemble while adaptively assigning greater importance to the most reliable base learners.

After determining optimal weights, all base models were retrained on the full training dataset, and final predictions on the test set were obtained using the weighted ensemble. This approach allows the model to adaptively assign higher importance to more reliable classifiers, improving predictive performance and generalization compared to simple averaging methods.

### 2.5 Explainable artificial intelligence using SHAP

Although ensemble models provide superior prediction performance, their decision mechanisms often lack transparency. To address this limitation and identify the most critical biomarkers, Shapley Additive Explanations (SHAP) was applied to the XGBoost component of the ensemble (Lundberg and Lee, 2017). SHAP applies principles from cooperative game theory to calculate the contribution of each feature on individual prediction outcomes. A reference background dataset composed of 200 samples from the SMOTE balanced training distribution was used for SHAP computation. TreeExplainer in SHAP was used with interventional feature perturbation, which is optimized for tree-based models. SHAP values were computed for all features in the test dataset, and the mean absolute SHAP value for each miRNA was used to rank its global importance. SHAP analysis was performed to quantify the contribution of individual miRNAs to the predictions of the XGBoost model. SHAP values were interpreted as model-specific feature contributions and were not considered evidence of biological causality or mechanistic regulation.

### 2.6 In-silico functional annotation/pathway analysis

To investigate the functional relevance of the top 20 SHAP-ranked miRNAs DIANA miRPath v4.0 and miRDB was utilized (Chen and Wang, 2020; Tastsoglou et al., 2023). miRTarBase 2022 was selected as the validated target database from the DIANA miRPath v4.0 database for identifying the target genes associated with PC. miRDB was also employed to identify the target genes associated with PC (Li et al., 2025).We used miRBase v22.1 annotations for miRNAs (Griffiths-Jones, 2006; Huang et al., 2022). KEGG pathway analysis identified biological pathways significantly regulated by the selected miRNAs (Kanehisa and Goto, 2000).

## 3 Results

### 3.1 Differential expression analysis of c-miRNAs

After integrating the five GEO datasets (Table 1) and applying ComBat batch correction to remove nonbiological variance, differential expression analysis using limma revealed 148 significantly dysregulated c-miRNAs between pancreatic cancer (PC) and healthy serum samples. These miRNAs met the stringent criteria of absolute log2 fold change of at least two and adjusted *p* value less than 0.05, demonstrating strong statistical confidence. To assess the effectiveness of batch correction, boxplots of miRNA expression distributions were generated before and after ComBat adjustment (Supplementary Figure S5). Prior to correction, the integrated GEO datasets exhibited noticeable differences in expression distributions, reflecting batch-specific technical variation. Following ComBat correction, the distributions became more consistent across cohorts, indicating successful reduction of batch effects while preserving biologically relevant variability for downstream analyses.

The volcano plot (Supplementary Figure S1) provided a global visualization of all miRNAs profiled across 1,101 samples. The plot displayed a clear tri-partition of miRNAs into upregulated, downregulated, and nonsignificant clusters. A substantial proportion of miRNAs showed marked downregulation in pancreatic cancer serum, which is consistent with previous findings that tumor suppressive miRNAs often show reduced abundance in cancer (Zen and Zhang, 2012). Highly significant downregulated markers were positioned on the left side of the plot, characterized by elevated negative log10 adjusted *p*-values, whereas upregulated miRNAs appeared distinctly on the right.

The Principal Component Analysis (Figure 2) further supported the presence of strong biological separation between PC and control samples. PC1 accounted for 17.5% of the variance, and PC2 accounted for 14.6%. The samples formed two clearly separable clusters, with minimal overlap. Healthy controls clustered tightly in a low-variance region, while PC samples occupied a wider region, possibly reflecting the biological heterogeneity of pancreatic cancer. The separation indicates that c-miRNA expression contains robust disease-specific information that is preserved even after merging multi-cohort data. These findings agree with earlier reports showing that c-miRNA signatures can effectively distinguish cancer from noncancer physiological states (Brase et al., 2010; Gallo et al., 2012).

**Figure 2:**
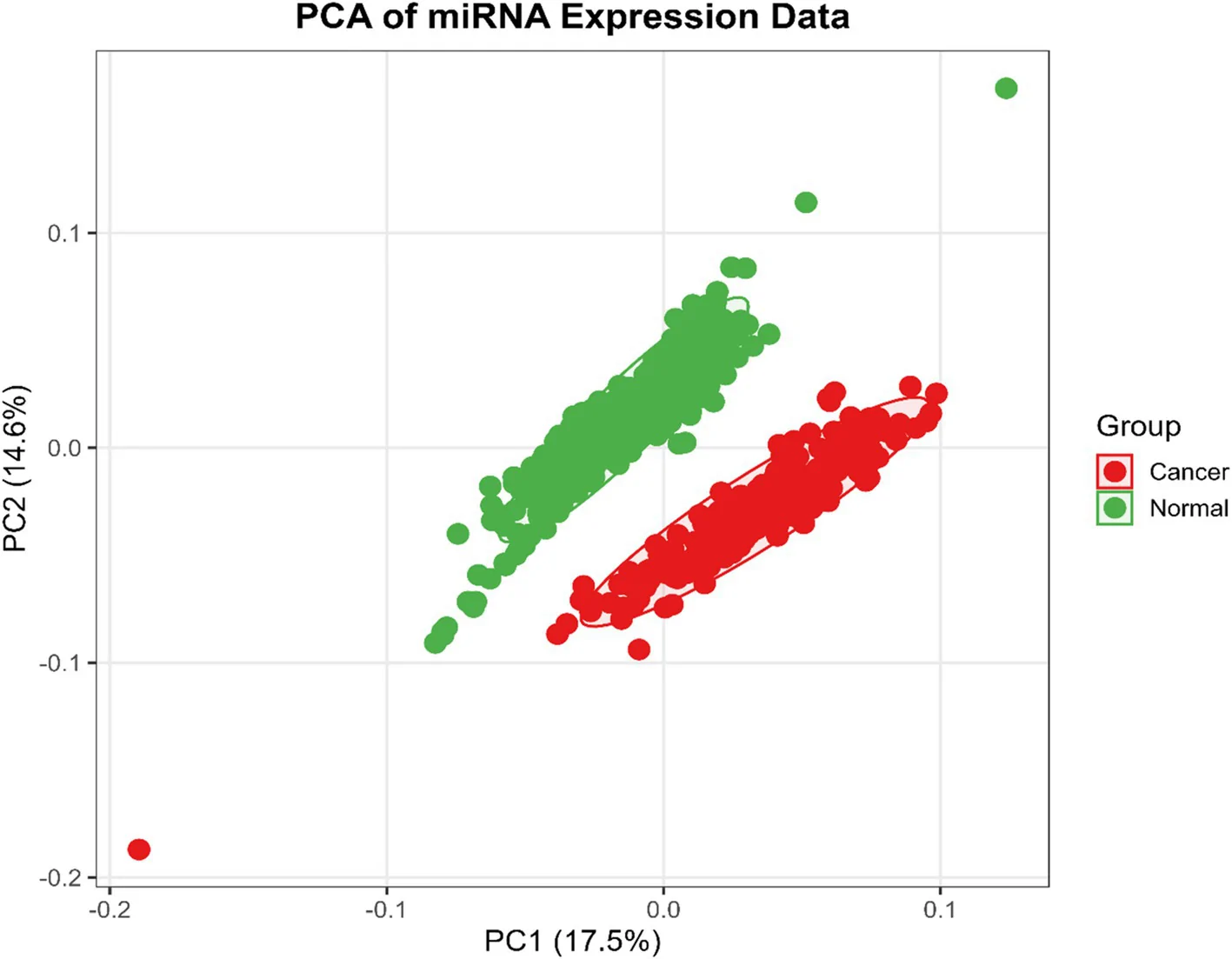
This principal component analysis (PCA) plot illustrates the separation of samples based on miRNA expression profiles. Each point represents an individual sample, color-coded according to group classification: normal (light green) and pancreatic cancer (maroon). The x-axis (PC_1_) explains 17.5% of the total variance, while the *y*-axis (PC_2_) accounts for 14.6%.

Together, the volcano and PCA analyses confirmed that batch-corrected c-miRNA profiles exhibit strong discriminatory potential, justifying their use in ML classification.

### 3.2 Machine learning classification and predictive performance

Following differential expression analysis, the 148 significant miRNAs were used to construct ML models aimed at distinguishing PC from healthy serum samples. After standardization and SMOTE oversampling to correct for class imbalance, five base classifiers were trained, including XGBoost, Random Forest, Support Vector Classifier, Naïve Bayes, and k-Nearest Neighbors. Their probability outputs were then aggregated using a Bayesian stacking framework, which automatically optimized classifier weights based on validation performance.

The Bayesian Stacked Ensemble model demonstrated highly robust performance on the independent test set of 221 samples. The model achieved an Accuracy: 97.28%, a Sensitivity (Recall): 94%, a Specificity: 99%, a Precision: 97%, and an F1 Score: 96% (Altman and Bland, 1994; Hanley and McNeil, 1982; Powers, 2011). The ROC curve (Figure 3) showed an early and steep ascent toward the upper left corner, indicating that the model maintained a high true positive rate even under very low false positive rates (Powers, 2011). The AUC of 0.97 demonstrated near-perfect discriminative capability. These findings align with previously reported diagnostic performance of serum miRNAs, where several studies have shown that miRNA signatures can outperform traditional biomarkers such as CA19-9 (Li et al., 2013; Wei et al., 2020).

**Figure 3:**
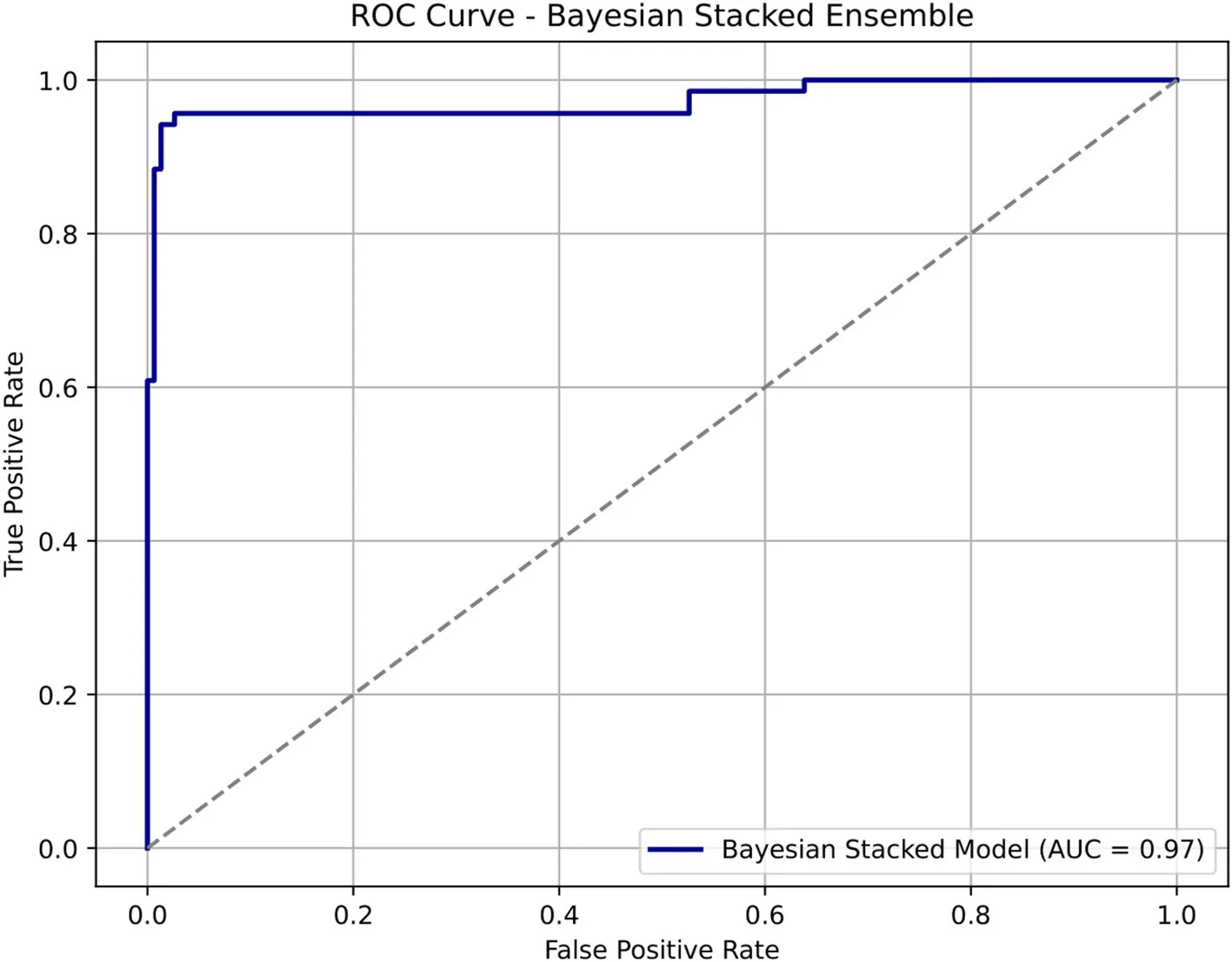
The ROC curve for the Bayesian stacked ensemble model showcases its better ability to distinguish between classes, with an almost perfect AUC (area under the curve) score of 0.97.

The results firmly indicate that integrating multiple classifiers through Bayesian stacking can effectively leverage complementary strengths of individual algorithms, leading to a highly reliable diagnostic model.

### 3.3 SHAP explainability reveals key predictive miRNAs

Although ensemble models offer strong predictive performance, interpretability is essential for biomarker discovery. Thus, SHAP analysis was applied on the XGBoost model to evaluate each miRNA’s individual contribution to the classification decision. The SHAP summary plot (Figure 4) highlighted the top 20 most influential miRNAs, which collectively had the strongest impact on model output. These miRNAs, along with their differential expression statistics, are listed in Table 2. The SHAP plot revealed both the magnitude and direction of influence for each miRNA across all samples.

**Figure 4:**
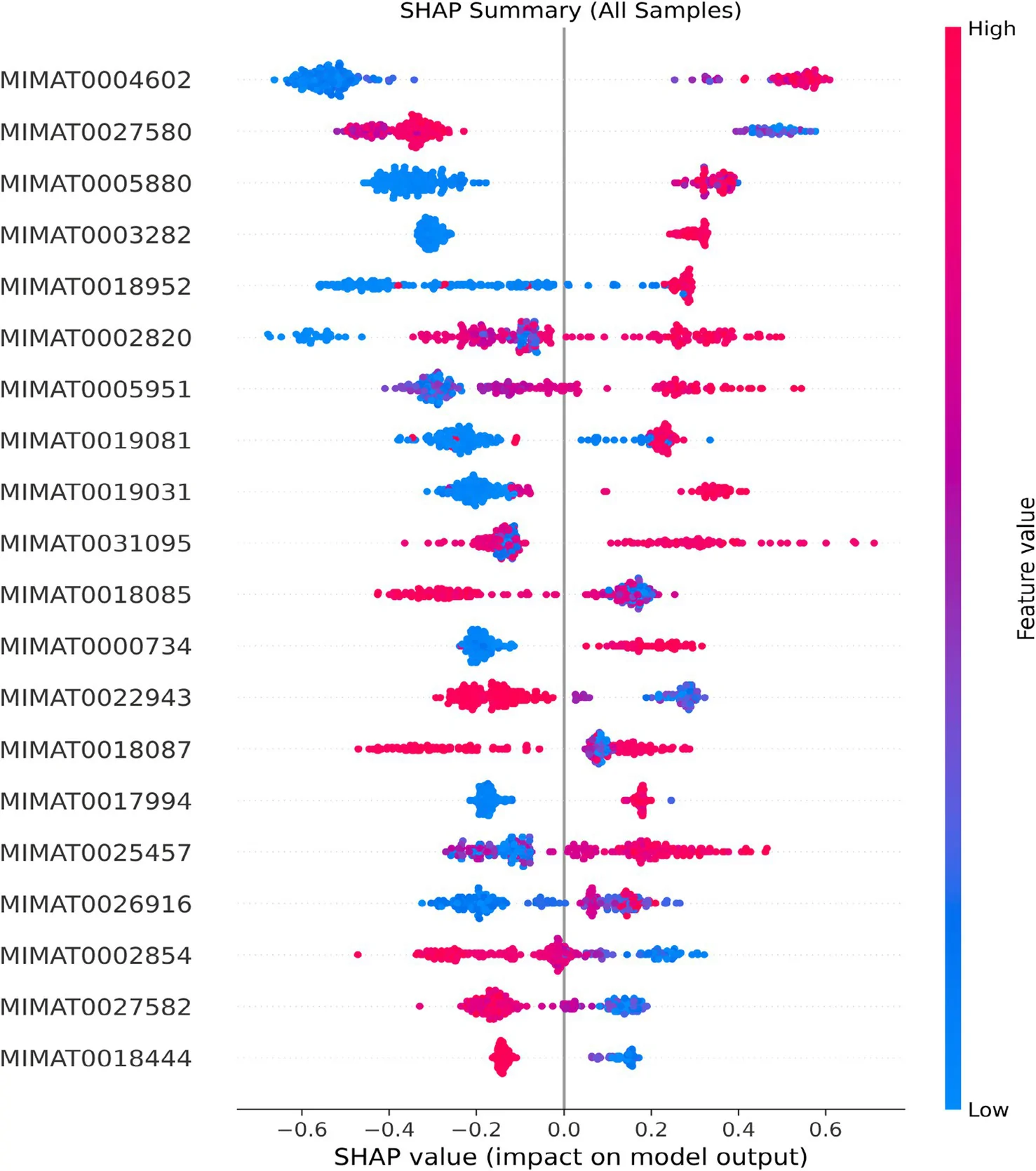
This SHAP (SHapley Additive exPlanations) summary plot visualizes the impact of the top 20 features, likely microRNAs on the model’s predictions.

**Table 2:** Top 20 c-miRNAs with their different values and regulation from SHAP.

S.no.	C-miRNAs	logFC	Adj. *p*. value	Regulation
1	hsa-miR-125a-3p	–2.944	<<0.001	Downregulated
2	hsa-miR-6839-5p	–2.134	<<0.001	Downregulated
3	hsa-miR-1290	2.774	<<0.001	Upregulated
4	hsa-miR-614	2.250	<<0.001	Upregulated
5	hsa-miR-4436a	–4.000	<<0.001	Downregulated
6	hsa-miR-497-5p	–2.665	<<0.001	Downregulated
7	hsa-miR-4538	–2.503	<<0.001	Downregulated
8	hsa-miR-128-2-5p	2.522	<<0.001	Upregulated
9	hsa-miR-4496	–2.482	<<0.001	Downregulated
10	hsa-miR-1307-3p	2.004	<<0.001	Upregulated
11	hsa-miR-3663-3p	2.483	<<0.001	Upregulated
12	hsa-miR-1233-5p	3.016	<<0.001	Upregulated
13	hsa-miR-3665	2.317	<<0.001	Upregulated
14	hsa-miR-380-5p	–2.424	<<0.001	Downregulated
15	hsa-miR-548az-3p	–2.498	<<0.001	Downregulated
16	hsa-miR-3615	–3.092	<<0.001	Downregulated
17	hsa-miR-1908-3p	–2.168	<<0.001	Downregulated
18	hsa-miR-521	–2.246	<<0.001	Downregulated
19	hsa-miR-6840-5p	–2.883	<<0.001	Downregulated
20	hsa-miR-642b-3p	2.394	<<0.001	Upregulated

hsa-miR-125a-3p emerged as the top-ranked miRNA. It showed strong downregulation (logFC −2.94) with extremely small *p* values. Its reduced serum expression consistently increased the predicted probability of pancreatic cancer. Earlier studies provide support for this observation through reports of altered levels of hsa-miR-125a-3p in PC and other cancers (Moriya et al., 2017; Seyed Salehi et al., 2022).

The dependence plot for hsa-miR-125a-3p (Supplementary Figure S2) illustrated a nonlinear inverse relationship between expression and SHAP value. Very low expression corresponded to strong positive contributions toward the “cancer” class, while higher expression tended to lower predicted disease probability. This effect demonstrates its potential utility as a risk stratification biomarker.

Other miRNAs with high SHAP importance included:

- hsa-miR-1290, previously reported as a powerful serum biomarker for early detection of pancreatic cancer (Li et al., 2013; Ta et al., 2018).
- hsa-miR-614, known to modulate cancer cell proliferation and associated with pancreatic and gastric cancer biology (Jing et al., 2021; Kabekkodu et al., 2020; Schultz et al., 2012).
- hsa-miR-497-5p, a tumor suppressive miRNA frequently downregulated in gastrointestinal cancers (Falzone et al., 2018; Liu et al., 2021).
- hsa-miR-3663-3p, previously associated with multiple cancer types and malignant biological processes (Sand et al., 2013; Yang et al., 2020).

The SHAP results demonstrate that the model’s predictions are biologically meaningful, rooted in miRNAs previously implicated in tumorigenesis, metastasis, drug resistance, and metabolic regulation.

### 3.4 Biological pathway validation of SHAP-ranked miRNAs

To evaluate significance the leading 20 SHAP-ranked miRNAs underwent pathway enrichment analysis utilizing DIANA miRPath v4.0 and miRDB. DIANA found 12 miRNAs with targets associated with signaling pathways relevant, to pancreatic cancer. These miRNAs controlled 23 genes altogether several of which play roles in cancerous mechanisms. Key targeted genes included CDK4, CDK6, CCND1, CDKN1A (cell cycle regulation), MAPK1, RAF1, MAP2K1 (MAPK signaling), TP53, BCL2L1, E2F3 (apoptosis and DNA damage response), and VEGFA, RELA (angiogenesis and inflammation). These pathways are well-established contributors to pancreatic carcinogenesis (Shams et al., 2020; Zhang et al., 2021). The KEGG pathway diagram (Supplementary Figure S3) showed that several of these miRNAs converge on classical pancreatic cancer pathways, reinforcing their biological validity. For instance, hsa-miR-497-5p targeted multiple components of the MAPK pathway, while hsa-miR-125a-3p regulated CDK4 and VEGFA, each of which is essential for cell proliferation and angiogenic signaling.

miRNAs not present in DIANA, such as hsa-miR-1290, hsa-miR-614, hsa-miR-128-2-5p, hsa-miR-548az-3p, hsa-miR-3615, and hsa-miR-6840-5p, were evaluated through miRDB, where predicted targets included genes involved in cytoskeletal remodeling, ubiquitin pathways, transcriptional regulation, and metabolic adaptation (Delfino and Rodriguez-Zas, 2013; Nonaka et al., 2022). The integrated results from DIANA and miRDB (Supplementary Table S1) support the hypothesis that the c-miRNAs identified by the ML model are biologically coherent markers involved in pathways central to pancreatic cancer initiation, progression, and metastasis.

## 4 Discussion

This study developed an integrative, reproducible pipeline that combines multi-cohort serum miRNA profiling, rigorous preprocessing and batch correction, differential expression analysis, an ensemble ML classifier with Bayesian stacking, and SHAP-based explainable artificial intelligence for identifying c-miRNA biomarkers for pancreatic cancer (PC). Utilizing five GEO datasets and a combined cohort of 1,101 serum samples in total, we identified 148 significant differentially expressed miRNAs and a panel of 20 miRNAs prioritized by SHAP for model interpretability and biological plausibility (see Tables 2 and Figure 4). The final Bayesian stacked classifier attained an AUC of 0.97, with 94% sensitivity and 99% specificity on the held-out test set, providing evidence that c-miRNA signatures can achieve clinically relevant discrimination between PC and healthy controls.

### 4.1 Comparison with prior c-miRNA studies in pancreatic cancer

Numerous studies have investigated c-miRNAs or specific miRNA panels as markers, for PC. Li et al. identified serum miR-1290 as a biomarker that distinguished early-stage PC from controls. Demonstrated that miR-1290 surpassed CA19-9 (carbohydrate antigen 19-9) is the only FDA-approved blood-based biomarker routinely used for pancreatic cancer (PC) management, including diagnosis, monitoring treatment response and detecting recurrence), in certain assessments (Arabkari et al., 2020; Li et al., 2013). Also Kojima et al. (2015) confirmed miRNA candidates and found that miR-125a-3p demonstrated high sensitivity for detecting pancreatic and biliary cancers within their study groups. In a study conducted by Mazza et al. 26 miRNAs were expressed differentially in tumors as compared to normal tissues in PC (Mazza et al., 2017). Later research and biomarker identification efforts uncovered candidates like miR-1246, miR-1290, miR-497-5p, among others consistently appearing in PC serum or plasma (Ganepola et al., 2014; Wei et al., 2020). Meta-analyses and systematic reviews confirm that c-miRNA panels generally demonstrate robust diagnostic performance and that combining miRNA signatures with CA19-9 improves overall accuracy (Pei et al., 2017; Peng et al., 2021; Wnuk et al., 2023). Our observation that hsa-miR-1290 and hsa-miR-125a-3p are among the top predictive features is therefore consistent with prior clinical and molecular studies, which increases confidence in the biological relevance of our findings (Li et al., 2013; Kojima et al., 2015; Seyed Salehi et al., 2022).

Although earlier studies produced promising candidate miRNAs, several methodological limitations are common: small sample sizes, single-cohort designs, limited handling of batch effects, reliance on single statistical tests without ML validation, lack of robust procedures for class imbalance, and limited interpretability of predictive models. The present study addresses many of these gaps in the following ways.

First, the combined cohort size of 1,101 serum samples, drawn from five GEO studies, substantially increases statistical power and reduces the chance of cohort-specific overfitting relative to single study reports. Multi-cohort aggregation is recognized as essential for producing robust biomarker candidates that generalize across populations and experimental conditions (Pei et al., 2017; Peng et al., 2021). Second, the use of empirical Bayes ComBat batch correction explicitly addresses inter-study technical variability, which can otherwise confound differential expression and model training. Many earlier biomarker studies did not comprehensively correct for batch effects or did so only within single studies, which reduces their cross-study reproducibility. By applying ComBat before differential expression and ML modeling, the study prioritized biological signal over technical noise, enabling more trustworthy feature selection (as shown by clear group separation in the PCA, Figure 2). Third, the analytic pipeline combined classical statistical differential expression (limma) with an advanced ML ensemble. While limma identified 148 significant DEMs with stringent thresholds (|log2FC| ≥ 2 and adj. *p* < 0.05), the Bayesian stacking approach integrated complementary strengths of five base classifiers (XGBoost, Random Forest, SVC, Naïve Bayes, k-NN), and optimized their weights via likelihood minimization. Ensemble stacking, particularly with constrained weight optimization, often improves calibration and generalization compared to single learners or naive averaging, and our model’s AUC of 0.97 highlights the efficacy of this strategy. Prior miRNA classification studies often reported single-algorithm performance or simpler combination rules; our approach benefits from rigorous out of sample testing and probabilistic weight optimization. This methodological synthesis represents a meaningful advancement for reproducible biomarker modeling. Fourth, class imbalance is a recurrent problem in biomarker datasets. The present work used SMOTE on the training partitions, which reduces bias toward majority classes and lowers the risk of inflated performance metrics that are common in imbalanced training regimes. Several earlier studies reported high AUCs but did not fully document resampling or class imbalance corrections, making their results hard to compare. The present approach documents SMOTE usage and assesses robustness by introducing a small proportion of label noise, enhancing the real-world credibility of reported metrics. Fifth, and critically, interpretability was built into the pipeline. The application of SHAP to the XGBoost model provided both global rankings of miRNA importance and local explanations for individual predictions. Interpretability is essential for clinical translation because clinicians require transparency about why a model flags a sample as high risk. Recent works that integrate explainable AI confirm this trend and show that SHAP-based explanations can reveal biologically plausible drivers that align with experimental evidence (Almisned et al., 2025; Hajjar et al., 2025). The integration of SHAP-based explainability represents a key strength of the proposed framework. Unlike conventional black-box models, SHAP provides quantitative estimates of feature importance, enabling identification of the circulating miRNAs that contribute most strongly to classification decisions. This not only improves model interpretability but also facilitates biological understanding and prioritization of candidate biomarkers for future mechanistic and clinical validation. Our SHAP results recovered known miRNAs such as miR-1290 and miR-125a-3p while also proposing additional candidates not consistently reported before. This simultaneous focus on prediction accuracy and feature explainability distinguishes our work from many earlier studies that prioritized either discovery or prediction alone.

Finally, in silico functional annotation was performed to provide biological context for the top SHAP-ranked miRNAs. Cross-referencing with DIANA miRPath and miRDB identified potential involvement in MAPK, CDK, and p53-related pathways. These analyses provide evidence of biological plausibility and generate hypotheses regarding potential miRNA–target interactions; however, they do not constitute experimental validation or establish a mechanistic role in pancreatic cancer (Shams et al., 2020; Zhang et al., 2021). Supplementary Table S1 summarizes the predicted and/or reported miRNA targets, including genes such as CDK4, CCND1, TP53, and VEGFA. Experimental validation using independent patient cohorts and orthogonal molecular approaches such as RT-qPCR and functional assays will be required to confirm these candidate biomarkers and their biological roles.

### 4.2 Biological interpretation and specific miRNA commentary

hsa-miR-125a-3p and hsa-miR-1290 were identified as the two significant miRNAs in our analysis. The consistency of hsa-miR-125a-3p and hsa-miR-1290 with findings from previous studies supports their prioritization as candidate circulating miRNA biomarkers. However, their recurrence across studies does not by itself establish causality, mechanistic involvement, or clinical validity. Similarly, the identification of hsa-miR-614 and hsa-miR-497-5p in our model provides candidates for further investigation, while their reported associations with cancer-related pathways should be considered hypothesis-generating rather than evidence of a mechanistic role in pancreatic cancer. hsa-miR-125a-3p showed consistent downregulation in our aggregated dataset and strong negative SHAP contributions when expressed at low levels. Previous studies have indicated a tumor suppressor function for miR-125a-3p. Have highlighted its diagnostic potential in various gastrointestinal cancers, such, as pancreatic and colon cancers (Kojima et al., 2015; Moriya et al., 2017; Wang et al., 2017). The consistency between our findings and independent experimental reports increases confidence that miR-125a-3p is a robust c-biomarker candidate.

hsa-miR-1290 has consistently been found at levels in the serum of PDAC patients and carries prognostic value surpassing CA19-9 across multiple cohorts (Li et al., 2013; Wei et al., 2020). The frequent identification of miR-1290 in studies and its high ranking in our SHAP analysis support its selection, for future clinical validation. Beyond these, miRNAs such as hsa-miR-614 and hsa-miR-497-5p that emerged in our model have mechanistic links to proliferation, apoptosis, and drug response pathways in other cancers. The combined evidence thus provides both predictive and mechanistic justification for experimental follow up. Importantly, SHAP-based feature importance should be interpreted as a measure of model behavior rather than biological causality. The high contribution of a miRNA to the model prediction does not establish that the miRNA drives pancreatic cancer development or represents a mechanistic biomarker. Correlations among miRNAs and other characteristics of the underlying datasets may also influence feature attribution. Therefore, the SHAP-prioritized miRNAs in this study should be regarded as computationally prioritized candidate predictive biomarkers requiring independent experimental and clinical validation.

### 4.3 Clinical and translational implications

The high AUC, combined with SHAP-driven interpretability and pathway validation, suggests the potential for a clinically actionable c-miRNA panel. Practical translation will require assay standardization, determination of clinically meaningful cut points, integration with existing clinical markers such as CA19-9, and prospective evaluation in high-risk populations or early detection cohorts. Prior meta-analyses indicate that combining c-miRNAs with CA19-9 improves diagnostic accuracy, a strategy that can be directly tested with the candidate miRNAs identified here (Peng et al., 2021). Additionally, explainable AI outputs can guide the selection of a minimal miRNA panel that retains high performance while reducing assay cost and complexity. A potential limitation of this study is that differential expression analysis was conducted prior to train-test partitioning, which may introduce a degree of information leakage from a strict ML perspective. However, this step was designed as a biological feature discovery process rather than a predictive feature selection procedure. Future work will incorporate nested cross-validation or fold-wise feature selection strategies to further validate the robustness of the identified biomarkers. Although the present study integrates five independent GEO cohorts to enhance robustness and reduce cohort-specific bias, it does not include a strictly independent external validation dataset. The multi-cohort design, combined with ComBat-based batch correction and evaluation on a held-out test set, partially addresses inter-study variability and supports generalizability. However, we acknowledge that validation on an entirely independent cohort would further strengthen the clinical applicability of the proposed model. Future work will therefore focus on incorporating prospective external cohorts to comprehensively evaluate model robustness across diverse populations. Another limitation is the clinical heterogeneity across the publicly available datasets. Although the five datasets provided a relatively large combined sample size, demographic and clinical variables were not uniformly reported or harmonized across studies. Consequently, factors such as disease stage, tumor subtype, patient characteristics, and treatment history could not be consistently incorporated into the analysis. Such unmeasured heterogeneity may influence circulating miRNA expression and contribute to variation in the identified predictive signature. Therefore, the present findings should not be interpreted as stage-, subtype-, or treatment-specific biomarkers. Future prospective studies using well-characterized cohorts with standardized clinical annotation will be required to assess the robustness of the candidate signature across clinically relevant patient subgroups. To facilitate reproducibility, detailed preprocessing procedures, model parameters, random seeds, computational settings, and additional methodological information have been provided in the Supplementary material, together with the relevant GEO accession numbers and computational workflow.

This research combines publicly available microarray datasets, resulting in variability in sample acquisition, patient characteristics, and technical handling that may not be completely addressed by batch correction. The retrospective nature of the datasets also limits control over clinical variables such as disease stage, tumor subtype, demographic characteristics, and treatment history, underscoring the need for prospective, well-annotated cohorts. Furthermore, the findings are based on microarray profiles and require validation using independent cohorts and orthogonal technologies such as RT-qPCR or digital PCR. Although SHAP enhances model interpretability by identifying predictive contributors, it remains a computational approach and cannot establish biological causality or replace experimental mechanistic validation. Similarly, the pathway and target analyses performed using public databases provide biological plausibility but do not constitute experimental validation. Finally, the model was developed to distinguish pancreatic cancer from healthy controls; therefore, clinical translation will require evaluation against benign pancreatic diseases and other clinically relevant conditions to determine diagnostic specificity.

## 5 Conclusion

This research introduces a multi-cohort computational approach that combines differential expression analysis, sophisticated ML and interpretable artificial intelligence to pinpoint c-miRNAs with high diagnostic value for pancreatic cancer. The aggregated dataset of 1,101 serum samples (from five GEO datasets) stringent batch correction and normalization. This procedure identified 148 miRNAs, from which a 20-miRNA panel was chosen. This panel showed classification performance indicated by an AUC of 0.97, in the Bayesian stacked ensemble model. SHAP-based interpretability highlighted biologically meaningful miRNAs such as hsa-miR-125a-3p and hsa-miR-1290, whose involvement in pancreatic cancer-related pathways was checked through DIANA miRPath and miRDB analyses. These findings affirm the clinical promise of c-miRNA panels as noninvasive biomarkers for facilitating early detection of pancreatic cancer. Future work should focus on experimental validation, prospective cohort evaluation, and assay standardization to translate these computationally derived biomarkers into clinically deployable diagnostic tools.

## Data Availability

The original contributions presented in the study are included in the article/Supplementary material, further inquiries can be directed to the corresponding author.
