# Supplementary material for: Explainable artificial intelligence identifies circulating microRNA candidate biomarkers for liquid biopsy-based pancreatic cancer detection
Source: Front Artif Intell. 2026 Oct 21;9:1810551. doi: 10.3389/frai.2026.1810551 (PMC13760841; doi:10.3389/frai.2026.1810551)
Supplement: Supplementary file 1 [file Data_Sheet_1.DOCX]

| **S. No.** | **Circulating miRNAs** | **Target Gene** | **Targets Resource** |
| --- | --- | --- | --- |
| 1 | hsa-miR-1233-5p | E2F3, TP53, BCL2L1, CDKN1A | miRTarBase 2022 |
| 2 | hsa-miR-125a-3p | CDK4, VEGFA | miRTarBase 2022 |
| 3 | hsa-miR-1307-3p | MAPK1, CDK4 | miRTarBase 2022 |
| 4 | hsa-miR-3663-3p | CDK6, CDKN1A | miRTarBase 2022 |
| 5 | hsa-miR-3665 | RAC1, CDKN1A | miRTarBase 2022 |
| 6 | hsa-miR-380-5p | CCND1 | miRTarBase 2022 |
| 7 | hsa-miR-4436a | CDKN1A, RELA | miRTarBase 2022 |
| 8 | hsa-miR-4496 | E2F3 | miRTarBase 2022 |
| 9 | hsa-miR-4538 | E2F3, E2F2, E2F2 (Pseudo gene) | miRTarBase 2022 |
| 10 | hsa-miR-497-5p | RAF1, MAP2K1, CCND1, RPS6KB1, E2F3, VEGFA, SMAD3, PIK3R1, CDK4, CDK6, AKT3, AKT3, MTOR, IKBKB | miRTarBase 2022 |
| 11 | hsa-miR-642b-3p | CDKN1A | miRTarBase 2022 |
| 12 | hsa-miR-6839-5p | BAK1 | miRTarBase 2022 |
| 13 | hsa-miR-1290 | SGO1, RTKN, HTRA3 | mirDB |
| 14 | hsa-mir-614 | EPB41L5 | mirDB |
| 15 | hsa-miR-128-2-5p | GNG13, CBFA2T3 | mirDB |
| 16 | hsa-miR-548az-3p | ZFHX3, CDC14A | mirDB |
| 17 | hsa-miR-3615 | KCTD12 | mirDB |
| 18 | hsa-miR-1908-3p | METTL21A | mirDB |
| 19 | hsa-miR-521 | NEU3 | mirDB |
| 20 | hsa-miR-6840-5p | FBXL19 | mirDB |

**Explainable Artificial Intelligence Identifies Circulating microRNA Biomarkers for Liquid Biopsy-Based Pancreatic Cancer Detection**

Saurav Mandal ^1^*, Md Aamir Ansari ^2^, Waribam Pratibha Chanu^2^

^1^Metabolomics, Proteomics and Imaging Division, Regional Medical Research Centre, Indian Council of Medical Research (ICMR), Dibrugarh, India

^2^Department of Bioinformatics, Central University of South Bihar, Gaya, India

^3^School of Vocational Studies and Applied Sciences, Gautam Buddha University, Greater Noida, Uttar Pradesh, India

*Correspondence: saurav.mandal@icmr.gov.in

**Supplementary Table 1:** Containing 20 circulating miRNAs with their target genes, which are involved in PC and other cancers.

**Supplementary Table 2:** Base Model Parameters

| **Model** | **Algorithm** | **Parameter** | **Value / Setting** | **Purpose** |
| --- | --- | --- | --- | --- |
| **xgb_clf** | XGBoost Classifier | n_estimators  max_depth  learning_rate  subsample  colsample_bytree  reg_lambda  use_label_encoder  eval_metric  random_state | 200  5  0.05  0.7  0.7  1.0  False  'logloss'  42 | Number of trees  Maximum tree depth  Step size shrinkage  Row sampling ratio  Column sampling ratio  L2 regularization term  Avoid legacy encoding  warning  Optimization metric  Reproducibility |
| **rf_clf** | Random Forest Classifier | n_estimators  max_depth  min_samples_split  min_samples_leaf  random_state | 200  5  5  2  42 | Number of trees  Maximum tree depth  Minimum samples to split  Minimum samples at leaf  Reproducibility |
| **svc_clf** | Support Vector Classifier | probability  kernel  C  gamma  random_state | True  'rbf'  1.0  'scale'  42 | Enables probability  estimates  Radial Basis Function  Regularization parameter  Kernel coefficient scale  Reproducibility |
| **nb_clf** | Gaussian Naive Bayes | *None specified* | Default | Uses standard normal  distribution |
| **knn_clf** | K-Nearest Neighbors | n_neighbors  metric | 5  'minkowski' | Number of neighbors  Distance metric (Euclidean equivalent) |

**Supplementary Figure 1:** The volcano plot provides a comprehensive visualization of differentially expressed miRNAs, highlighting their regulation patterns based on statistical significance and fold change. The plot is divided into three main categories: downregulated (left side), not significant (central region), and upregulated (right side) miRNAs. The y-axis represents the Log10 Adjusted P-Value, indicating the statistical significance of the expression changes, with higher values denoting greater significance. The x-axis shows the Log₂ Fold Change, where negative values correspond to downregulation and positive values to upregulation.

**Supplementary Figure 2:** The dependence plot illustrates the dependence of SHAP values for the miRNA MIMAT0004602, providing insights into how specific features influence the model's predictions. The y-axis represents the SHAP values, which quantify the impact of each feature on the output, with positive values indicating a higher likelihood of the predicted outcome and negative values suggesting a reduced likelihood. The x-axis represents the values of the features, illustrating their distribution and connection to the SHAP values.

**Supplementary Figure 3**: The pathways of pancreatic cancer show us that 7 different pathways are involved.

```
Python version: 3.12.7 | packaged by Anaconda, Inc. | (main, Oct  4 2024, 13:17:27) [MSC v.1929 64 bit (AMD64)]
Detailed info: sys.version_info(major=3, minor=12, micro=7, releaselevel='final', serial=0)

Package         | Version
------------------------------
numpy           | 1.26.4
scipy           | 1.13.1
scikit-learn    | 1.5.1
pandas          | 2.2.2
xgboost         | 2.1.4
shap            | 0.46.0
matplotlib      | 3.9.2
combat          | 0.3.3
conda           | 24.11.3
xgboost         | 2.1.4
```

**Supplementary Figure 4:** Python Packages and versions used for reproducibility

**Supplementary Figure 5:** Comparison of miRNA expression distributions before and after ComBat batch correction
